# Uptake of gold nanoparticles in HeLa cells observed by confocal microscopy shows dependency on particle size and shape

**DOI:** 10.1007/s00249-025-01769-5

**Published:** 2025-06-19

**Authors:** Kimiya Pakravanan, Virginia Bazzurro, Marco Salerno, Alberto Diaspro

**Affiliations:** 1 Dipartimento di Fisica, Università di Genova, Genoa, Italy; 2 Nanoscopy and NIC@IIT, CHT, Istituto Italiano di Tecnologia, Genoa, Italy

**Keywords:** Living cells, Gold nanoparticles, Confocal microscopy, Nanoparticles uptake, Green fluorescent protein, Viability assay

## Abstract

Uptake of gold nanoparticles by HeLa cells fixed at different incubation times of up to eight hours was investigated using 3D confocal laser scanning microscopy followed by image analysis. The cell bodies were characterized by fluorescence labeling, whereas the gold nanoparticles were identified by light scattering. The amount of nanoparticles uptaken at different times was evaluated with Fiji according to a dedicated protocol. We assessed the effect of particle size (80 and 150 nm diameter spheres) and shape (spheres vs “urchins”) on their uptake. The large spherical nanoparticles presented approximately fourfold higher levels of uptake than the nanourchins. Also, the spheres presented a clearly increasing uptake in the time profile, reaching a maximum around seven hours and then showing a slight decrease. We ascribe this behavior to a lower aptitude of the cells for taking up nanoparticles with either urchin shape or smaller size and to the possible presence of competing kinetics for exocytosis at the longest times. Live experiments confirmed for the time profile a relatively flat cell response to the urchins and an increasing response to the spheres, yet with a final plateau. However, in the live case, the internalization levels were as low for the spheres as for the urchins.

**Supplementary Information:**

The online version contains supplementary material available at 10.1007/s00249-025-01769-5.

## Introduction

Understanding the interactions of biological systems with nanoscale materials, typically nanoparticles (NPs), is of interest for preventing the occurrence of diseases associated with exposure to these materials (e.g., inflammation of lung and heart, adverse effects in organs, cancer) as well as for diagnosing and treating existing diseases (e.g., use as drug delivery carriers, thermal or magneto-thermal therapy with metal particles) (Murthy 2007; Nikzamir et al. 2021). In practical terms, the interaction of living cells with NPs means mainly their uptake and internalization, which occurs via endocytosis (Iversen et al. 2011; Manzanares and Ceña 2020; Rennick et al. 2021; De Almeida et al. 2021). As the model nanomaterial of interest in this work, we selected gold NPs (AuNPs) (Dykman and Khlebtsov 2014). AuNPs are generally recognized to be biocompatible (Kus-liśkiewicz et al. 2021) even though some researchers have also pointed out possible risks of toxicity (Alkilany and Murphy 2010). In any case, they represent a promising option for biomedical applications such as drug delivery—being prone to functionalization via thiol chemistry—and cancer photothermal therapy—being prone to heat absorption (Hammami et al. 2021). In some cases, AuNPs for photothermal therapy could also be chemically synthesized inside the cells proceding from gold salt (Schwartz-Duval et al. 2020). AuNPs can also play a significant role in angiogenesis (Darweesh et al. 2019), involved in disease conditions of either reduced or excess vascularity (tissue ischemia or cancer). Still another application of AuNPs on target cells is in transfecting them with small interfering RNA (Yue et al. 2017). Of course, interaction of AuNPs with cells, both for uptake and subsequent action, is strongly affected by the possible functionalization or coating, which is often based on polyethyleneglycol (Deng et al. 2019; Magogotya et al. 2022; Fernández et al. 2015), phospholipids (Shearer and Petersen 2019), peptides (Liu et al. 2007), mannose ligands (Le Guével et al. 2015) or even inorganic materials such as e.g. iron oxide (Kolosnjaj-Tabi et al. 2015). On the side of the living cells, we decided to adopt the HeLa cell line, which is also a common model in biology, thanks to their robust nature and wide availability (Nott 2020; Masters 2002).

To adequately describe and characterize the AuNPs uptake by living cells, techniques that can both quantify the number of AuNPs that entered the cells and possibly reveal their localization and interaction with the cells are highly desirable. However, many of the works in the current literature were based on mass spectrometry measurements, which allows only for indirect counting of the number of AuNPs inside the cells based on their size, (see e.g. (Wang et al. 2019; Chithrani et al. 2006; Xie et al. 2017; Ma et al. 2025)). Other AuNP detection methods are optical in nature yet do not provide direct imaging, being based on plasmon (Wang et al. 2010) or SERS effect (Kapara et al. 2020; Sujai et al. 2020) or image correlation spectroscopy (Katoozi et al. 2021). On the other hand, compared to imaging techniques that provide higher resolution yet require sample conditions that are more distant from native ones, such as electron microscopy, optical microscopy is still the technique of choice for imaging samples of living cells (Diaspro and Bianchini 2020). By integrating in our imaging protocol both fluorescence detection—for the cell structures—and reflection-mode detection—for the AuNPs—we could obtain a label-free 3D mapping of the distribution of AuNPs around and inside the cells after different incubation times in culture (in vitro), at diffraction-limited resolution. We report on the application of our method to the quantitative analysis of cellular uptake of commercial AuNPs having different sizes and morphology, namely 80 nm “urchins” and 80 and 150 nm spheres.

## Experimental

### Cell culture

HeLa cells previously transfected to express a green fluorescent protein (GFP) were used. Cell culture was grown in 90% Dulbecco's Modified Eagle's Medium–high glucose (DMEM, Sigma-Aldrich, St. Louis, MO, USA, product No. D5796), 10% fetal bovine serum (Sigma-Aldrich, St. Louis, MO, USA, product No. F9665), 2.0 mM glutamine (Sigma-Aldrich, St. Louis, MO, USA, product No. G7513), 100 IU/mL penicillin–streptomycin (Sigma-Aldrich, St. Louis, MO, USA, product No. P4333) and maintained in a humidified atmosphere of 95% air and 5% CO_2_ at a temperature of 37 °C. For the experiments, cells were plated on poly-L-lysine-coated (Sigma-Aldrich, St. Louis, MO, USA, product No. P2636) glass coverslips, at the density of 5.0–10 × 10^4^ cells on 18 mm diameter coverslips for uptake studies and 1.0–2.0 × 10^5^ cells on 25 mm diameter coverslips for cell health assays.

### Incubation with AuNPs and cell labeling

Commercial AuNPs were obtained from Sigma-Aldrich (150 nm spheres, product No. 746649, and 80 nm urchins, product No. 797723) and BBI-Solutions (80 nm spheres, product No. EM.GC80). The Sigma NPs were described as “reactant-free”, stabilized suspension (pH 6–8) in 0.1 mM phosphate buffered saline (PBS), with particle concentration of ~ 3.6 × 10^9^ mL^−1^ for the spheres, ~ 7.8 × 10^9^ mL^−1^ for the urchins. The BBI NPs were described as having a citrate capping agent with a particle concentration of 1.1 × 10^10^ mL^−1^. The AuNPs were characterized by dynamic light scattering (DLS) for both size and Zeta potential, by means of a Zetasizer nano (Malvern Panalytical, Malvern, UK). The results are reported in Fig. S1.

The cell incubation time with bare AuNPs was at a constant concentration for varying durations of typically 1, 2, 3, 4, 5, 6, 7, and 8 h. Only in some cases, they were incubated also for either shorter (0.25 and/or 0.5 h) or longer (24 h) times, for details see the Results section. On the day of the experiment, HeLa cells were incubated at 37 °C with AuNPs diluted at different ratios to compensate for the different stock solution concentrations, namely 1:1000 for the 150-nm spheres, 1:2200 for the urchins, 1:3000 for the 80-nm spheres. The samples were then washed twice in Dulbecco's PBS (DPBS, Sigma-Aldrich, St. Louis, MO, USA, product No. D8662) and labeled with Cell Mask Deep Red at a concentration of 1:1000 (Thermo Fisher Scientific, product No. C10046). After 30 min incubation at 37 °C, the cells were washed twice with DPBS and post-fixed with Formaldehyde solution 4%, buffered, pH 6.9 (Sigma-Aldrich, St. Louis, MO, USA, product No. 1.00496) for 15 min at room temperature, washed twice with DPBS, and finally mounted on optical microscope glass slide (1″ × 3″) with ProLong^™^ Glass Antifade (Thermo Fisher Scientific, product No. P36980).

### Imaging

A Stellaris8 (Leica Microsystems, Mannheim, Germany) inverted confocal laser scanning microscope was used to acquire the images. A white light laser provided the desired excitation wavelengths, and avalanche photodiodes were used as detectors. The samples were imaged through a plan-apochromatic oil immersion objective 63 ×/1.40 NA. The standard format adopted for the single-layer images was 1024 × 1024 pixels, with a 16-bit scale for the light intensity. The number of layers in the 3D confocal stack was in the range of 20–30, depending on the local sample thickness, with a layer z spacing of ~ 0.3 μm. The lateral scan size (field of view) changed from region to region, depending on the cell density, in the 50–70 µm range, such that the pixel (linear) size was around 70–50 nm. The pinhole was set to 1.00 AU (Airy unit). The incident laser intensity was set to 2% of the available maximum that is estimated to be of the order of 1 mW. Two fluorescence channels were acquired in transmittance mode, setting the exciting laser line wavelength at 638 nm for Cell Mask Deep Red control labeling and at 488 nm for the GFP expressed by the HeLa cells, with collecting windows of 648–705 nm and 498–557 nm, respectively. The scattering signal from the AuNPs was collected in reflection mode, with an incident laser line at 580 nm wavelength and a 560–600 nm collecting window. The channels were acquired frame-by-frame across the different settings, with a typical line scan frequency of 200 Hz and line-averaging set to 2, resulting in an acquisition time of around 20 min for each stack.

### Image analysis protocol

The freeware software Fiji (v1.54f) (Schindelin et al. 2012), a powered distribution of ImageJ (Abràmoff et al. 2004), was used, with plugins *3D Objects counter* (Bolte and Cordelières 2006) and *3D manager* from *3D Suite* (Ollion et al. 2013). The LIF project file obtained from the Leica program LasX was opened and the three channels (C1: AuNPs in reflection, C2: expressed GFP labeling all cell volume, C3: Cell Mask Deep red for cell membrane) were separated by command *Image*—*Color*—*Split channels*. The AuNPs and GFP cell image stacks were low-pass filtered to remove the possible effect of noise (*Process*—*Filters*—*Gaussian blur 3D*, with 1 sigma width selected for all 3 directions) and duplicated (*Image*—*Duplicate*, *duplicate stack*) to be able to restart processing easily in case of mistakes. For the AuNPs analysis, the command *Analyze*—*3D OC options* was selected and the options *Centroid* and *Integrated density* were checked to evaluate the occurrence of possible aggregates through intensity analysis (see Discussion). After selecting *Analyze*—*3D Objects Counter* on the AuNPs image stack, the layer where most AuNPs appeared was selected (typically, the glass coverslip z level, for which AuNPs are out of the cells borders) and the *Threshold* was set to make the mask select most NPs without exceeding merging of some of them. The Size filter Min was set to 0 (with Max set to the highest possible value, in order not to avoid considering possible aggregates), and Exclude objects on edges was not checked. The maps of *Objects* and *Centroids*, and the options *Statistics and Summary* for reporting, were selected. In the text window *Log*, the number of resulting objects appeared. The list of object properties (mainly, the x,y, and z positions of the centroids) appeared as a table in the text window *Statistics* for Channel-filename-series name.

Then, the objects were imported to the dialog of *3D Manager* after first setting the options for measurements by this plugin, under *Plugins*—*3D Suite*—*3D Manager Options*. The options *Objects numbering*, *Centroid*, *Volume,* and *Surface* were selected (the latter two for the subsequent cells after AuNPs analysis). *Sync with selected * *3D viewer* was selected and *Drawing * was set to Contour. Then *Plugins*—*3D Suite*—*3D Manager* was selected. After selecting the image of *Centroids*, *Add image* was clicked. The list of objects (AuNPs) appeared in the empty field. *Select all* was clicked and each object was renamed with the base name AuNP to avoid possible ambiguity later during the processing. Then the same process was done for the cells, i.e., C3 of the image: precautionally, *Image*—*Duplicate*; *Process*—*3D Gaussian filter*; *Analyze*—*3D Objects Counter*. Particularly in this case, the best mask did not have to exclude the edges (the cells certainly touched some for all images). Yet, excluding some small “cell” objects (fragments thereof, setting Min size to e.g. 1000) was convenient. A few objects were thus obtained, representing integer cells or their pieces (for cells crossing the image boundaries). More objects could be excluded by increasing the Min size. A summary list of all information on treatment parameters for the given 3D stack image was annotated—the most important being the two segmentation thresholds used for AuNPs and cells—in an Excel spreadsheet. This time the *Object map* of C3 was selected. In the *3D manager dialog*, *Add image* was clicked, the new objects added to the list were selected and renamed as Cell. With the *Cell objects* selected, *Live ROI* was clicked so that it toggled from *Off* to *On*. When *Measure 3D* was clicked, the measurements of the cell objects were obtained, among which *Volume* and *Surface*. When the image of *AuNPs centroids* was selected, and in the *3D Manager* dialog *Quantif 3D* was clicked, a new table appeared, one of the last columns being Nb. Objects (i.e., AuNPs) inside any given object in the first column (i.e., ROI, cell object). Summing them all in the Excel sheet gave the total number of AuNPs contained in the cells. The previous information about the cells objects (Volume and Surface) was used in one Excel sheet to calculate AuNP concentration (per unit volume or area) or the number of AuNPs inside the cells normalized to the total number of AuNPs in the field of view.

### Statistical analysis

In a given experiment, for each AuNP type and incubation time, at least three images were acquired and analyzed (sample size *N* ≥ 3). The quantities of interest (mainly the number of AuNPs inside the cells divided by the respective total cell volume) were averaged, and the mean was considered for plotting in the results (see Fig. 2) with error bars as large as the population standard deviation. To evaluate the statistical difference between the data points, we carried out one-way ANOVA with post-hoc Tukey’s pair tests for different levels of significance α of 5% (pair marked *), 1% (pair marked **) and 0.1% (pair marked ***). ANOVA was done on SigmaPlot 12.0 (Grafiti LLC, Palo Alto, CA, US).

### Cell health assays

MTT (3-[4,5-dimethylthiazol-2-yl]−2,5 diphenyl tetrazolium bromide) was purchased from Sigma-Aldrich, St. Louis, MO, USA (product No. M2128). This assay is based on the conversion of MTT salt into purple formazan crystals by living cells, which determines mitochondrial dehydrogenase activity and provides cell viability. HeLa cells were exposed to 150 nm spherical AuNPs and 80 nm gold urchins for various incubation times (spanning from 1 to 8 h), followed by treatment with 0.25 g/L MTT in the culture medium without serum for 2 h at 37 °C. After removing the medium, the resulting formazan crystals were dissolved in dimethyl sulfoxide, and cell viability was assessed by measuring absorbance using a UV–Vis spectrophotometer (Cary3000, Agilent) at 570 nm wavelength in disposable polystyrene cuvettes (see Tables S1 and S2 in the Supplementary Information).

Live/Dead assay (Thermo Fisher Scientific, product No. L3224) was carried out on HeLa cells cultured on 25 mm poly-L-lysine coated coverslips under similar conditions as those used for the AuNPs counting experiments at similar incubation times. Then, imaging was performed over large areas, and the numbers of live (green) and dead (red) cells were manually counted across multiple frames. This analysis resulted in an average total cell count of 1.2 × 10^4^ for 150 nm AuNPs and 1.3 × 10^4^ for 80 gold nm urchins (refer to Tables S3, S4, and Fig. S5 in the Supplementary Information).

## Results

**Fig. 1:**
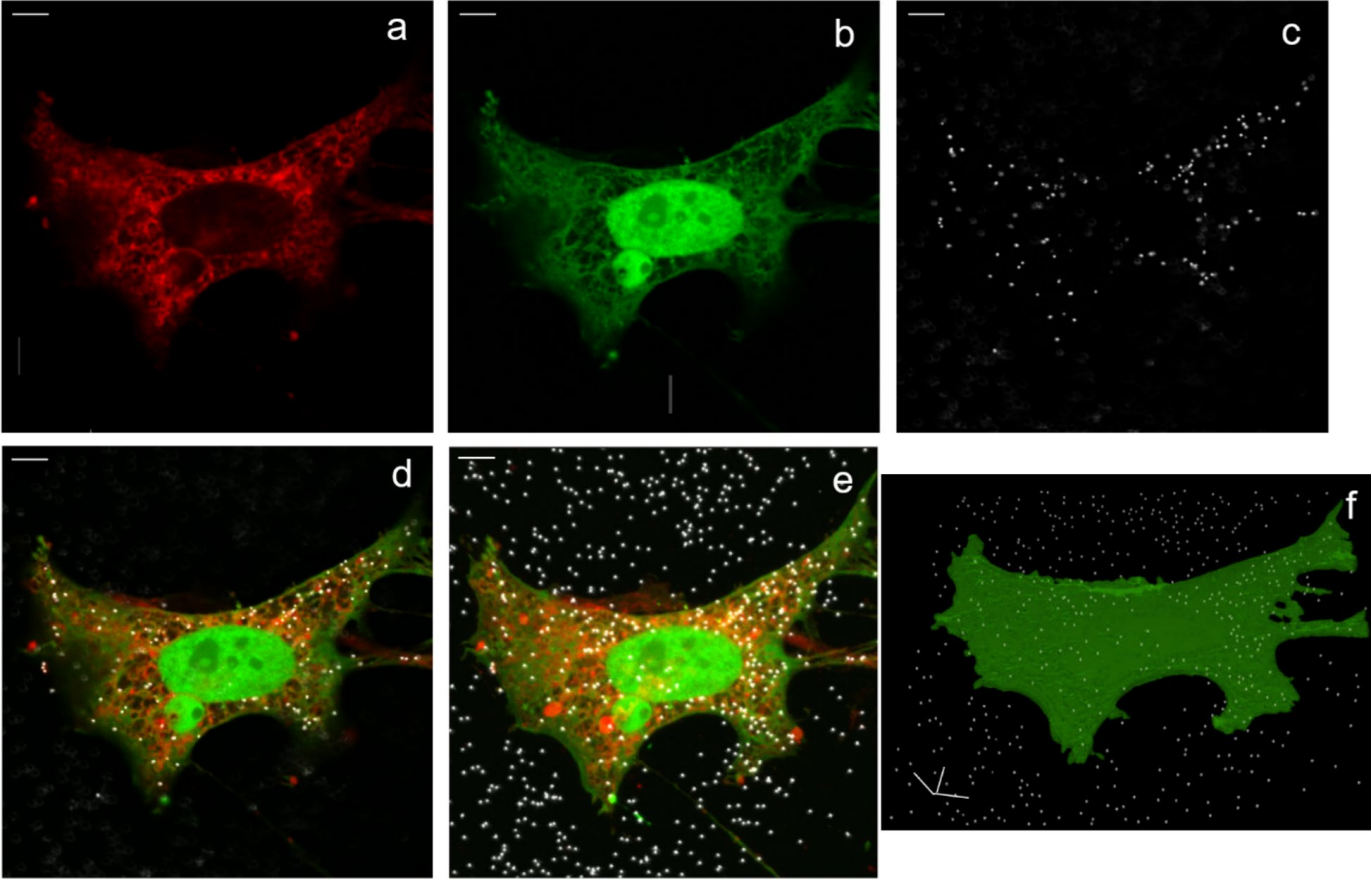
Typical image of HeLa cell exposed to AuNPs (here, one case of 150 nm spheres incubated for 6 h). **a**–**c** The three separated channels at an intermediate height layer; red is the cell membrane (Cell Mask Deep Red), green is the whole cell (GFP), white is the AuNPs. **d** overlay of the three channels in (**a**–**c**). **e** 2D projection of the three overlaid channels across all heights: **f** 3D overlay of cell (green) and AuNPs centroids (white) obtained in Fiji (all scale bars are 5 μm long)

An example of the 3D images obtained in this study is shown in Fig. 1. The pixel size set for our images was always in the range of 50–70 nm. These values correspond to considerable oversampling when the Nyquist-Shannon rule is considered, that the pixel size should be at least as small as half the possible resolution (Diaspro and van Zandvoort 2021), which for diffraction-limited optical microscopy in the visible can be assumed ~ 250 nm. However, it should be considered that some low-pass filtering was to be applied later on, during image processing and analysis (see Section"Image analysis protocol").

Figure 1a–d shows the three distinct channels (plus the overlay) at a single intermediate z position in the stack. Figure 1e shows the 2D projection of all the layers in the stack (with the three channels overlaid). This latter view captures all the AuNPs (white spots), including those inside the cell boundaries and those sitting on the glass coverslip substrate outside the cell. This is different from the single intermediate layer sliced in Fig. 1d, where all the appearing AuNPs are actually inside the cell. Notice that in no case were there AuNPs inside the cell nucleus, due to the relatively large size, different from other studies on ultrasmall AuNPs (Huo et al. 2014; Sokolova et al. 2022). In Fig. 1f, the 3D view obtained with Fiji is shown, where the AuNPs are rendered as their dimensionless centroid points only. Here, we avoided rendering the cell membrane (red channel) and set the cell (green channel) to 70% transparency. These settings make it easier to appreciate the AuNPs positions inside the cell and avoid any unclear assignment, see more in the Discussion. The difference with the whole AuNPs bodies can be seen clearly when the latter ones are plotted instead, in white, as in the original microscope rendering program, see Fig. S2. The steps between layers in Fig. 1f have been left intentionally unsmoothed. A better perception of the 3D distribution in this 3D stack is possible when viewing an animation of the data during 360° rotation around one axis passing through the intermediate layer, see Supplementary Information.

**Fig. 2:**
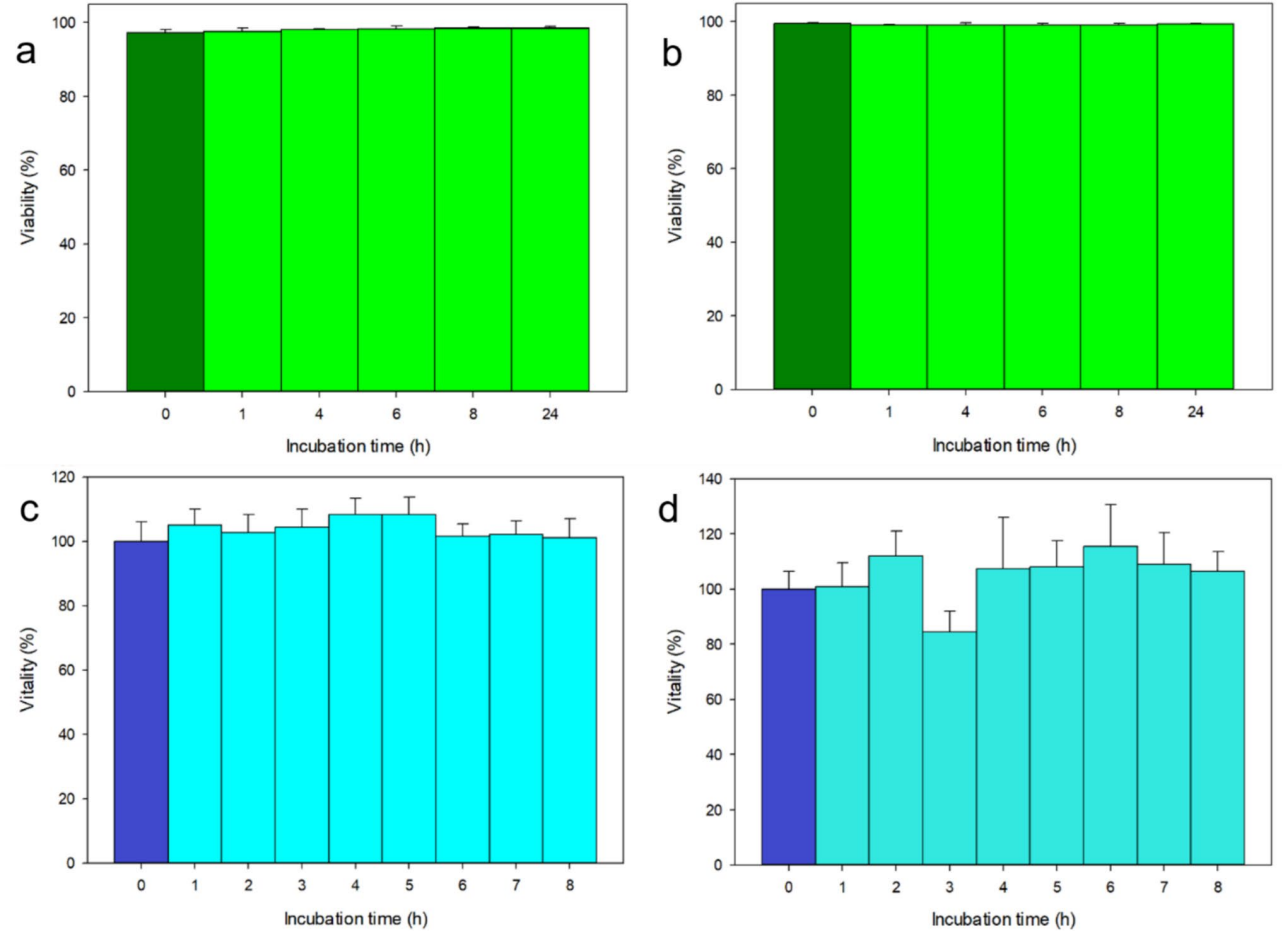
Cell condition assays for HeLa cells exposed to different AuNPs. **a**, **b** Live/dead viability for exposure to **a** 150 nm spheres and **b** 80 nm urchins; **c**, **d** MTT vitality for exposure to **c** 150 nm spheres and **d** 80 nm urchins

Before analyzing the amount of AuNPs uptaken by the HeLa cells at different times, we wanted to assess the possible adverse effect of the former by standard assays. In Fig. 2, we present the results of Live/Dead (top row, Fig. 2a, b) and MTT assays (bottom row, Fig. 2c, d) for the case of 150 nm spheres (left column, Fig. 2a, c) and the 80 nm urchins (right column, Fig. 2b, d).

In all cases shown in Fig. 2, no significant cytotoxic effect of exposure to AuNPs appeared, confirming the expected biocompatibility of these nanomaterials, after the literature (Kus-liśkiewicz et al. 2021; Hammami et al. 2021). For the MTT of 80 nm urchins (Fig. 2d), fluctuations appeared with lower values down to below 80%, apparently occurring especially at longer times; however, no statistically significant difference emerged among all groups (*N* = 4).

**Fig. 3:**
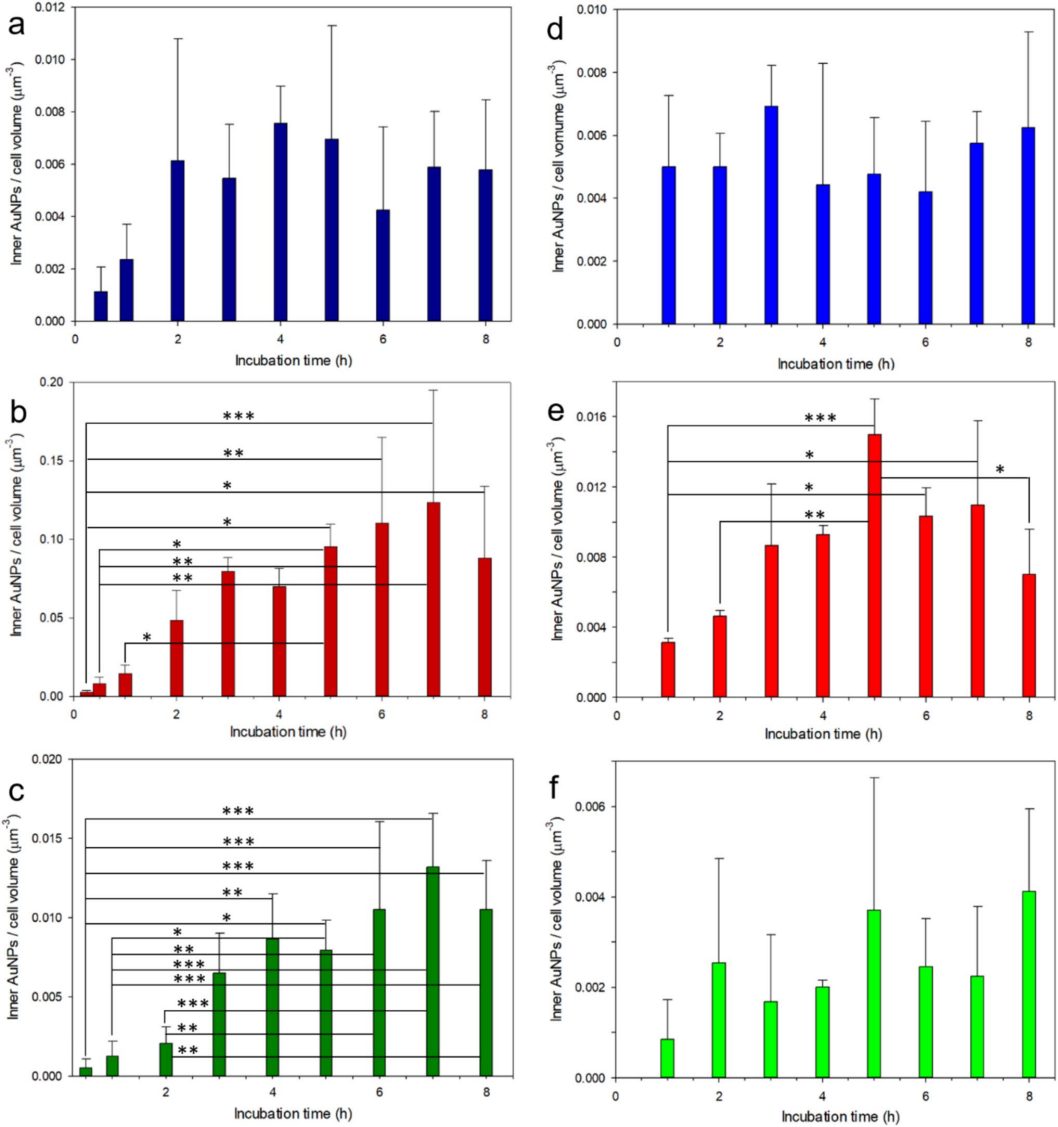
Time profiles of AuNPs uptake by the HeLa cells. **a**–**c** (left column): cells fixed after AuNPs incubation, **d**–**f** (right column): cells exposed to AuNPs and imaged while in culture (living) without fixation. **a**, **d** 80 nm diameter urchins, **b**, **e** 150 nm diameter spheres, **c**, **f** 80 nm spheres. * statistically significance difference according to 95%, ** according to 99%

Based on the image analysis presented in Fig. 1c, using the cell as 3D ROIs and counting the AuNPs centroids inside them, the quantitative information summarized in Fig. 3 was obtained, which is the main result of this work, along with the proposed methodological approach. It should be noted that, different from Fig. 2 where the x-axis times are labels describing only qualitatively the different values, in Fig. 3 we spaced them according to the respective quantitative values, to let the eye have a guide along the possible underlying time profile.

In the left column panels of Fig. 3, the results obtained for the three different types of AuNPs investigated are presented, in the case of incubation and fixation of cells at various times before imaging (*N* = 4). Figure 3a shows the time-profile sequence obtained for the 80 nm urchins. Whereas a slightly increasing trend may be seen as underlying the heights of the bars, the highest value of all appears at a relatively short time (4 h). Overall, given the low absolute values at different times and the relatively large error bars (representing the standard deviations), the behavior is such that we may interpret these differences as fluctuations independent of a real response of the system. Indeed, ANOVA showed no significant difference between any pairs of values reported in this graph, not even for the first two data points at the shortest time (*p* = 0.054).

When we compare Fig. 3a with 3b, that is one experiment done with 150 nm diameter spheres, we can see instead, in the second case, a time profile with a clearly increasing number of uptaken AuNPs. Actually, an anomalous local minimum appears at an intermediate time (4 h), which deviates from the increasing trend, showing all the way up to 7 h. However, this difference with the surrounding values at 3 h and 5 h does not appear to be statistically significant. At 8 h, finally, a decrease is apparent with respect to the 7 h peak value. In this experiment, ANOVA pointed out statistically significant differences for several pairs of groups between the initial—and lowest—values and the final—and highest—ones; for details, see Fig. 3b.

In Fig. 3c, the case of round (spherical) AuNPs with 80 nm diameter is shown. Again, an overall increasing trend with time appears, similar to the case of larger spheres in Fig. 3b, despite the size being as small as for the urchins in Fig. 3a, with some statistically significant differences (see stars in the figure).

In addition to the different trends over time (increasing for the spheres until longer times while soon saturating for the urchins), the absolute numbers of concentrations of uptaken AuNPs should also be considered, at least as to the orders of magnitude. Apparently, the highest values for the 150 nm spheres are in the order of 0.1 AuNPs per µm^3^ (see left scale in Fig. 3b), whereas they are much smaller, roughly one order of magnitude less (~ 0.01 AuNPs per μm^3^) for the 80 nm urchins (Fig. 3a). For the spheres as small as the urchins (also 80 nm diameter, Fig. 3c), most values are also around 0.01 / μm^3^, with only one as high as almost three times as much (the maximum at 7 h).

In the right column panels of Fig. 3, similar experiments for the case of living cells are shown. It can be seen that a similar qualitative behavior shows up, with an increasing trend with time for the spherical AuNPs (Fig. 3e and f) and a rather flat response with random fluctuations for the urchins (Fig. 3d). Similarly, the different entity of the absolute amount of uptaken AuNPs (lowest for urchins, highest for large spheres and intermediate for small spheres) reflects the qualitative results obtained for the case of fixed cells after AuNPs incubation up to the given time point.

**Fig. 4:**
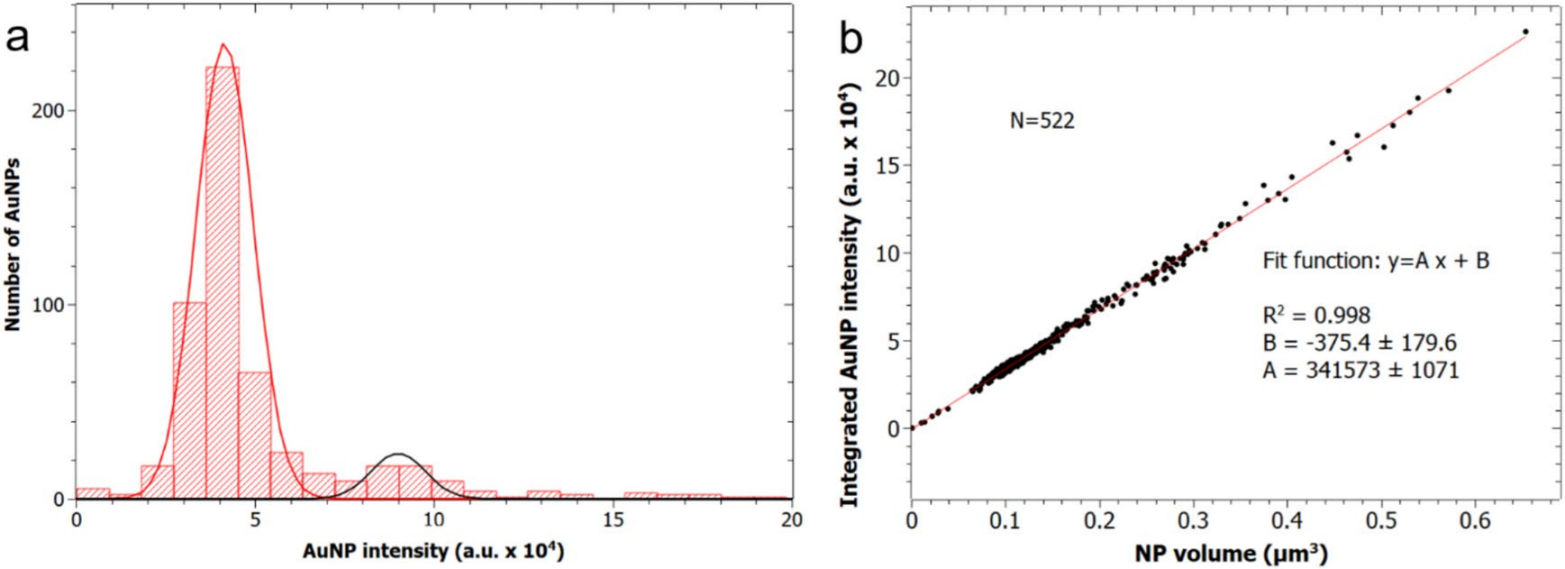
Exemplary AuNPs aggregation analysis (150 nm spheres, 6 h incubation time, image 3 of 4). **a** Histogram representing the distribution of intensities in a given typical scattering image of AuNPs, **b** plot showing the correlation between scattered light intensity as obtained from the relevant plugin (parameter Integrated Density) and the apparent volume of the respective AuNP “object”

One possible issue in our AuNPs identification could be the relevant presence of aggregates. In a similar work, the occurrence of NPs aggregates was evaluated by carrying out higher-resolution imaging via STED (Torrano et al. 2013). Since we used light scattering instead of fluorescence for detecting our AuNPs, we could not perform STED on them. However, in a few random cases, we carried out intensity analysis of the respective images to evaluate the amount of possible AuNP aggregates. It was assumed that the light intensity scattered by AuNPs aggregates should be a multiple of that resulting from single—separated—AuNPs. The results of a similar test are presented in Fig. 4. Figure 4a shows the occurrence of different intensity populations, which have been fitted with Gaussian profiles. The dominant peak at the lowest intensity—centered at ~ 4.3 × 10^4^ a.u.—corresponds to the population of primary AuNPs. In addition to that, only another single peak emerges, located at twice as much the intensity of the previous one, ~ 8.8 × 10^4^ a.u. Assuming a linear dependence of scattering intensity from the AuNPs with their volume, and neglecting shape-effects in first approximation, this minor peak is obviously due to agglomerates of two AuNPs. On the right-hand side, some more counts appear, too few to define additional peaks, yet consistent with the presence of aggregates of three and even four AuNPs (occurring at three- and four-fold intensity). Considering the proportions of the areas under the two peaks and outside, we conclude that in this case over a total of 522 AuNPs counted, 88% were primary particles and 8% were binary aggregates, a reasonable deviation from the ideal case of no aggregation. Figure 4b confirms the linear correspondence between AuNP volume and scattered light intensity. The peak value in Fig. 4a corresponding to the single AuNPs apparently arises from AuNPs with a volume of 0.12 μm^3^, namely diameter around 500 nm, which is roughly consistent with the diffraction-limited resolution size of primary AuNPs of either type (80 or 150 nm real diameter).

## Discussion

Concerning the proof of biocompatibility of our AuNPs, we have to mention the limitation that the Live/Dead and MTT tests presented in Fig. 2 do not include the case of small AuNP spheres. Whereas a priori we can’t be sure that such a case does not differ from the others, that was actually our assumption in the present study. For the Live/Dead assay, we notice that imaging was carried out until 24 h since AuNPs incubation, i.e., well beyond the maximum incubation time of 8 h used for quantitative analysis of uptake. On the other hand, sampling at shorter incubation times was more sparse than for MTT, which, in turn, describes not only the cell survival but also their metabolic functionality, which is more relevant and was sampled with similar time points as those used for the uptake experiment. In Fig. 2a (MTT vitality of cells incubated with 150 nm spherical AuNPs), it should be noted that even values higher than the 100% control (no AuNPs incubation) appeared. The reasons can be the intrinsic variability in the number of cells incubated in the different wells of the multiwell plate and the effect of cell proliferation. The latter, additionally, cannot be ascribed in higher amount to the longest incubation times for the AuNPs, as the living cells were not synchronized in their cycle with the beginning of incubation. Therefore, this effect may appear, and is random, also in cells incubated for a time shorter than that of the normal life cycle duration (~ 24 h) of human cells, such as HeLa.

For NPs in cells, super-resolution approaches have also been used recently, which appear to be helpful when either identification of precise subcellular compartments is requested (Sheth et al. 2023) or trafficking is investigated (Van Der Zwaag et al. 2016). In the future, we also aim to use higher resolution imaging and tracking, possibly by means of the Minflux technique (Salerno et al. 2025), for example focusing on the AuNPs induced genotoxicity (Magogotya et al. 2022). However, in this preliminary study we just aimed at assessing the number of uptaken AuNPs, for which identifying the cell boundaries and assigning the NPs inside those bodies was sufficient, a task that was efficiently carried out by confocal microscopy only.

The label-free AuNPs detection method based on scattering was inspired by former work (Gusta et al. 2024; Sheth et al. 2023). Here, we combined it with a novel image analysis protocol specifically aimed at characterizing the time profile uptake of the selected living cell model. We used the same Fiji plugin 3D Object Counter as in (Torrano et al. 2013), but—different from their approach—we did not separately identify the membrane region but only split the space into inner and outer areas with respect to the cell membrane. We think it somewhat arbitrary to define a membrane thickness in the micrometer range (typically 1.4 µm in Torrano et al.). At the same time, it is known that the lipid bilayer should be around 4 nm, a value much smaller than even the smallest AuNPs used here (80 nm diameter). Instead, for the unambiguous assignment of the AuNPs to either inner or outer space concerning the cell membrane, we evaluated the dimensionless point-like position of the AuNPs via their centroids, provided by the 3D Suite plugin (see Section"Image analysis protocol"). Other researchers, similar to (Torrano et al. 2013), defined a cell surface region and assigned some uptaken AuNPs to that (Wang et al. 2010). It should be noted that, in our approach, those AuNPs “on the cells” would be classified as “out” instead of “almost” or “close” to”in”.

The major advantage of analyzing the phenomenon of endocytosis by light microscopy is exactly that one of being close to native conditions, different from e.g., electron microscopy, which allows for imaging even in live conditions. We remind that this capability was also used for the Live/Dead assay, as shown in Fig. 2. Actually, in the case of live imaging the statistics was lower, *N* = 3 instead of *N* = 4 (as for Fig. 3a–c). The reason was the acquisition time of the confocal microscope, taking approximately 20 min per image: being the time-points between 1 and 8 h spaced of 1 h each, not more than 3 images could be acquired around the considered time-point (assuming them all representative of the same central time value: i.e., for example, image taken starting at 40 min, 60 min and 80 min, all being inserted into the “bin” of the 1 h = 60 min time-point). For the same reason, we had to skip the shortest time points below 1 h (0.25 and 0.5 h), which were occasionally observed in the case of fixed cells. It should be mentioned that the given size for the urchins corresponds to an effective diameter thereof, for which the urchin tips (needles) take no more than 20% of the total (Moustaoui et al. 2019).

Part of the results in Fig. 3 on the fixed cells was repeated (150 nm spheres and 80 nm urchins only) and is shown in Fig. S3. Also in that case, a trend of increasing internalization with incubation time appeared for the spherical AuNPs (with some statistically different values). For the live cells only, one additional intermediate size of the spherical AuNPs was investigated, namely 100 nm diameter, which is shown in Fig. S5, and apparently confirms again the increasing uptake with incubation time, even if with the large fluctuations of the live case (*N* = 3 only). In contrast, the time response for the nanourchins was relatively flat (no statistically significant differences). It should be noted here that a difference between the shortest exposure time (0.5 h, where present) and the other times (≥ 1 h) is somewhat trivial when occurring, as, of course, in the limit of shortest exposure time (eventually zero) the AuNPs that entered the cells also must be very low (eventually zero as well), so the most relevant statistically significant differences are only those emerging on a longer exposure.

When comparing our results with those in the literature, we can see that most studies of NPs uptake over time deal with different NP materials. For example, Torrano et al. (Torrano and Bräuchle 2014) investigated the uptake of small (8 and 30 nm diameter) ceria NPs by HMEC-1 cells, up to much longer incubation times than we did (72 h) and also observed a maximum number of internalized NPs at an intermediate time of around 24 h, which was ascribed to cell division. In the same work, for much larger silica NPs (310 nm), a study of times up to 24 h also found a maximum at 24 h, but with a sharp increase only between 4 and 10 h, which would be consistent with our maximum at around 7 h. In Blechinger et al. (2013), the uptake of 310 nm silica NPs by HeLa cells was investigated, and the authors concluded that the number of NPs in the cells depended more on the cell type rather than the incubation time. In particular, HeLa cells were much less prone to uptaking silica NPs than healthy (non-disease) HUVEC cells. AuNPs uptake was studied in the works by Chithrani et al. (2006) and (Xie et al. (2017). The former authors investigated the effect of both size and shape of the AuNPs on their uptake into mammalian cells, specifically HeLa same as in our work. They observed reaching a plateau, rather than a maximum, at around the same time point as we did, namely ~ 7 h. This result is more similar to that one of our experiments in live cells (Fig. 3, right column). Actually, the size of their AuNPs was on average lower than ours, being all spherical with a diameter between 14 and 74 nm. It should be noted that, concerning this size, the maximum absolute number of uptaken AuNPs was observed at an intermediate size of 50 nm diameter. A similar result was observed by Ko et al. with adipose-derived stem cells (Ko et al. 2015). As for the shape different from spherical, rods were investigated, with aspect ratios of 1:3 and 1:5, which resulted in lower uptake than 1:1 spherical AuNPs. This result is qualitatively consistent with our for urchins, differing from spheres and showing less uptake. In the work of (Xie et al. 2017), the uptake of AuNPs in the form of rods, stars, and triangles was investigated in RAW264.7 cells at 8 and 24 h. The highest levels of internalization were observed with triangular AuNPs and the lowest with star-like ones. Also, in this case, we see that the shape closest to the symmetric sphere case (the triangle) appeared to be more keen on being uptaken by the cells, with respect to shapes exhibiting more sharp tips around their edge. It should be noticed that their AuNPs were, in all cases, coated with methyl-polyethylene-glycol to obtain an electrically neutral charge, for excluding interference of factors other than shape itself. Our AuNPs were not coated, other than the possible presence of coordinated ions from the colloidal solution containing diluted salt, which was PBS for the spheres and citrate for the urchins, aiming to stabilize them. In fact, when we measured the Zeta potential (see Fig. S1), we found for our spherical AuNPs values above 30 mV for the absolute potential, indicating the absence of significant aggregation, as also observed after casting and possible uptake by the cells, see Fig. 4. The nanourchins appeared borderline in this respect, with a Zeta potential of − 25 mV, corresponding to a size distribution not monodispersed but elongated towards larger hydrodynamic size (polydispersity index ~ 0.3 vs < 0.1 for the nanospheres), which can be due to the peculiar shape. The importance of the surface charge of AuNPs in uptake by cells has been pointed out in several works. For example, Lunnoo et al. pointed out the effect of surface charge in AuNPs translocation through the plasma membrane of mammalian cells carrying out also molecular dynamics simulations (Lunnoo et al. 2019), while Srijampa et al. showed that different charge elicited different endocytosis mechanisms in human leukaemic monocyte lymphoma cell lines U937 (Srijampa et al. 2019). From our side, future work, which will focus on a specific biological question instead of the method, should certainly use AuNPs with the same stabilization medium, to avoid side effects of different electric charges affecting the comparisons between different AuNPs. As to the effect of AuNP size and shape on the endocytosis mechanism, Xie et al. investigated that using different inhibitors, concluding that all AuNPs with different shapes underwent clathrin-mediated endocytosis, while rods also utilized caveolae/lipid raft mediated endocytosis, and triangles uptake was associated with strong cytoskeletal rearrangement and dynamin pathway. We have not addressed the possible uptake mechanism so far, but this will likely be the subject of future work.

## Conclusion

Our study reported on a new methodological approach for quantifying the cellular uptake of gold nanoparticles (AuNPs) using 3D confocal laser scanning microscopy imaging, based on label-free scattering of AuNPs. The spherical 150 nm AuNPs showed significantly higher uptake than the 80 nm urchins, which generally agrees with the existing literature. It is likely that the more tip-shaped AuNPs represent for the cell membrane a tougher obstacle for internalization due to the higher membrane bending energy barrier to be overcome in this case. Whereas the spherical AuNPs exhibited an increasing time profile trend on internalization, reaching a maximum—for fixed cells—or a plateau—for live cells—around 7 h incubation time, the urchins showed a more irregular time profile, apparently flat, apart from random fluctuations (except the obviously lowest values at the very short time of 0.5 h). The case of fixed cells showing a slight decline at 8 h, could be due to competing exocytosis in parallel with ongoing endocytosis. Biocompatibility tests confirmed minimal cytotoxicity, supporting the biomedical application potential of AuNPs. During the revisions of the present manuscript a new research article appeared also based on the use of AuNP scattering for quantification of cellular uptake, via intensity changes in flow-cytometry (Shin et al. 2025), which confirms the interest of our approach.

## Supplementary Information

Below is the link to the electronic supplementary material.

Supplementary file1 (DOCX 1468 KB)

Supplementary file2 (AVI 11242 KB)

## Data Availability

The data that support the findings of this study are available from the corresponding author, MS, upon request.

## References

[CR1] Abràmoff MD, Magalhaes PJ, Ram SJ (2004) Image processing with image. J Biophotonics Int 11:36–42

[CR2] Alkilany AM, Murphy CJ (2010) Toxicity and cellular uptake of gold nanoparticles: what we have learned so far? J Nanopart Res 12(7):2313–2333. 10.1007/s11051-010-9911-8 21170131 10.1007/s11051-010-9911-8 PMC2988217

[CR3] Blechinger J, Bauer AT, Torrano AA, Gorzelanny C, Bräuchle C, Schneider SW (2013) Uptake kinetics and nanotoxicity of silica nanoparticles are cell type dependent. Small 9(23):3970–3980. 10.1002/smll.201301004 23681841 10.1002/smll.201301004

[CR4] Bolte S, Cordelières FP (2006) A guided tour into subcellular colocalization analysis in light microscopy. J Microsc. 10.1111/j.1365-2818.2006.01706.x 17210054 10.1111/j.1365-2818.2006.01706.x

[CR5] Chithrani BD, Ghazani AA, Chan WCW (2006) Determining the size and shape dependence of gold nanoparticle uptake into mammalian cells. Nano Lett 6(4):662–668. 10.1021/nl052396o 16608261 10.1021/nl052396o

[CR6] Darweesh RS, Ayoub NM, Nazzal S (2019) Gold nanoparticles and angiogenesis: molecular mechanisms and biomedical applications. Int J Nanomed. 10.2147/IJN.S223941 10.2147/IJN.S223941 PMC6756918 31571869

[CR7] De Almeida S, Mauro ES, Drasler B, Taladriz-Blanco P, Petri-Fink A, Rothen-Rutishauser B (2021) Understanding nanoparticle endocytosis to improve targeting strategies in nanomedicine. Chem Soc Rev: R Soc Chem. 10.1039/d0cs01127d 10.1039/d0cs01127d PMC8111542 33666625

[CR8] Deng L, Liu H, Ma Y, Miao Y, Xiaoli F, Deng Q (2019) Endocytosis mechanism in physiologically-based pharmacokinetic modeling of nanoparticles. Toxicol Appl Pharmacol. 10.1016/j.taap.2019.114765 31669777 10.1016/j.taap.2019.114765

[CR9] Diaspro A, Bianchini P (2020) Optical nanoscopy. Rivista Del Nuovo Cimento, Springer Science and Business Media Deutschland, Berlin

[CR10] Diaspro A, van Zandvoort MAMJ (2021) Super-resolution imaging in biomedicine. CRC, Boca Raton

[CR11] Dykman LA, Khlebtsov NG (2014) Uptake of engineered gold nanoparticles into mammalian cells. Chem Rev. 10.1021/cr300441a 24279480 10.1021/cr300441a

[CR12] Fernández TD, Pearson JR, Leal MP, Torres MJ, Blanca M, Mayorga C, Le Guével X (2015) Intracellular accumulation and immunological properties of fluorescent gold nanoclusters in human dendritic cells. Biomaterials 43(1):1–12. 10.1016/j.biomaterials.2014.11.045 25591956 10.1016/j.biomaterials.2014.11.045

[CR13] Gusta MF, Ernst LM, Moriones OH, Piella J, Valeri M, Bastus NG, Puntes V (2024) Long-term intracellular tracking of label-free nanoparticles in live cells and tissues with confocal microscopy. Small Methods. 10.1002/smtd.202301713 38564783 10.1002/smtd.202301713

[CR14] Hammami I, Alabdallah NM, Al Jomaa A, Kamoun M (2021) Gold nanoparticles: synthesis properties and applications. J King Saud Univ- Sci. 10.1016/j.jksus.2021.101560

[CR15] Huo S, Jin S, Ma X, Xue X, Yang K, Kumar A, Wang PC, Zhang J, Zhongbo Hu, Liang XJ (2014) Ultrasmall gold nanoparticles as carriers for nucleus-based gene therapy due to size-dependent nuclear entry. ACS Nano 8(6):5852–5862. 10.1021/nn5008572 24824865 10.1021/nn5008572 PMC4076024

[CR16] Iversen TG, Skotland T, Sandvig K (2011) Endocytosis and intracellular transport of nanoparticles: present knowledge and need for future studies. Nano Today. 10.1016/j.nantod.2011.02.003

[CR17] Kapara A, Brunton V, Graham D, Faulds K (2020) Investigation of cellular uptake mechanism of functionalised gold nanoparticles into breast cancer using SERS. Chem Sci 11(22):5819–5829. 10.1039/d0sc01255f 34094083 10.1039/d0sc01255f PMC8159335

[CR18] Katoozi D, Clayton AHA, Moss DJ, Chon JWM (2021) Uptake quantification of gold nanoparticles inside of cancer cells using high order image correlation spectroscopy. Biomed Opt Express 12(1):539. 10.1364/boe.417321 33659088 10.1364/BOE.417321 PMC7899503

[CR19] Ko WK, Heo DN, Moon HJ, Lee SJ, Bae MS, Lee JB, Sun IC, Jeon HB, Park HK, Kwon IK (2015) The effect of gold nanoparticle size on osteogenic differentiation of adipose-derived stem cells. J Colloid Interface Sci 438(January):68–76. 10.1016/j.jcis.2014.08.058 25454427 10.1016/j.jcis.2014.08.058

[CR20] Kolosnjaj-Tabi J, Javed Y, Lartigue L, Volatron J, Elgrabli D, Marangon I, Pugliese G et al (2015) The one year fate of iron oxide coated gold nanoparticles in mice. ACS Nano 9(8):7925–7939. 10.1021/acsnano.5b00042 26168364 10.1021/acsnano.5b00042

[CR21] Kus-liśkiewicz M, Fickers P, Tahar IB (2021) Biocompatibility and cytotoxicity of gold nanoparticles: recent advances in methodologies and regulations. Int J Mol Sci. 10.3390/ijms222010952 34681612 10.3390/ijms222010952 PMC8536023

[CR22] Le Guével X, Perrino MP, Fernández TD, Palomares F, Torres MJ, Blanca M, Rojo J, Mayorga C (2015) Multivalent glycosylation of fluorescent gold nanoclusters promotes increased human dendritic cell targeting via multiple endocytic pathways. ACS Appl Mater Interfaces 7(37):20945–20956. 10.1021/acsami.5b06541 26329370 10.1021/acsami.5b06541

[CR23] Liu Y, Shipton MK, Ryan J, Kaufman ED, Franzen S, Feldheim DL (2007) Synthesis, stability, and cellular internalization of gold nanoparticles containing mixed peptide-poly(ethylene glycol) monolayers. Anal Chem 79(6):2221–2229. 10.1021/ac061578f 17288407 10.1021/ac061578f

[CR24] Lunnoo T, Assawakhajornsak J, Puangmali T (2019) In silico study of gold nanoparticle uptake into a mammalian cell: interplay of size, shape, surface charge, and aggregation. J Phys Chem C 123(6):3801–3810. 10.1021/acs.jpcc.8b07616

[CR25] Ma J, Liu Y, Zhang L, Yao L, Ding Y, Qin H, Wang Z et al (2025) Size-dependent internalization of gold nanoparticles in individual tetrahymena thermophila characterized by single-cell mass cytometry. Environ Pollut. 10.1016/j.envpol.2025.126030 40064228 10.1016/j.envpol.2025.126030

[CR26] Magogotya M, Vetten M, Roux-van der Merwe MP, Badenhorst J, Gulumian M (2022) In vitro toxicity and internalization of gold nanoparticles (AuNPs) in human epithelial colorectal adenocarcinoma (Caco-2) cells and the human skin keratinocyte (HaCaT) Cells. Mutat Res - Genetic Toxicol Environ Mutagen. 10.1016/j.mrgentox.2022.503556 10.1016/j.mrgentox.2022.503556 36462792

[CR27] Manzanares D, Ceña V (2020) Endocytosis: the nanoparticle and submicron nanocompounds gateway into the cell. Pharmaceutics. 10.3390/pharmaceutics12040371 32316537 10.3390/pharmaceutics12040371 PMC7238190

[CR28] Masters JR (2002) HeLa cells 50 years on: the good, the bad and the ugly. Nat Rev Cancer 2(4):315–319. 10.1038/nrc775 12001993 10.1038/nrc775

[CR29] Moustaoui H, Saber J, Djeddi I, Liu Q, Movia D, Prina-Mello A, Spadavecchia J, Chapelle MLDL, Djaker N (2019) A protein corona study by scattering correlation spectroscopy: a comparative study between spherical and urchin-shaped gold nanoparticles. Nanoscale 11(8):3665–3673. 10.1039/c8nr09891c 30741295 10.1039/c8nr09891c

[CR30] Murthy SK (2007) Nanoparticles in Modern medicine: state of the art and future challenges. Int J Nanomed 2:129–141 PMC2673971 17722542

[CR31] Nikzamir M, Akbarzadeh A, Panahi Y (2021) An overview on nanoparticles used in biomedicine and their cytotoxicity. J Drug Deliv Sci Technol. 10.1016/j.jddst.2020.102316

[CR32] Nott R (2020) “HeLa Cell Line.” Arizona State University - Embryo Project Encyclopedia. 2020. https://keep.lib.asu.edu/items/173753

[CR33] Ollion J, Cochennec J, Loll F, Escudé C, Boudier T (2013) TANGO: a generic tool for high-throughput 3D image analysis for studying nuclear organization. Bioinformatics 29(14):1840–1841. 10.1093/bioinformatics/btt276 23681123 10.1093/bioinformatics/btt276 PMC3702251

[CR34] Rennick JJ, Johnston APR, Parton RG (2021) Key principles and methods for studying the endocytosis of biological and nanoparticle therapeutics. Nat Nanotechnol. 10.1038/s41565-021-00858-8 33712737 10.1038/s41565-021-00858-8

[CR35] Salerno M, Bazzurro V, Angeli E, Bianchini P, Roushenas M, Pakravanan K, Diaspro A (2025) MINFLUX nanoscopy: a ‘Brilliant’ technique promising major breakthrough. Microsc Res Tech. 10.1002/jemt.24765 39838620 10.1002/jemt.24765 PMC11972445

[CR36] Schindelin J, Arganda-Carreras I, Frise E, Kaynig V, Longair M, Pietzsch T, Preibisch S et al (2012) Fiji: an open-source platform for biological-image analysis. Nat Methods. 10.1038/nmeth.2019 22743772 10.1038/nmeth.2019 PMC3855844

[CR37] Schwartz-Duval AS, Konopka CJ, Moitra P, Daza EA, Srivastava I, Johnson EV, Kampert TL et al (2020) Intratumoral generation of photothermal gold nanoparticles through a vectorized biomineralization of ionic gold. Nat Commun. 10.1038/s41467-020-17595-6 32913195 10.1038/s41467-020-17595-6 PMC7483505

[CR38] Shearer LJ, Petersen NO (2019) Internalization of phospholipid-coated gold nanoparticles. Crystals. 10.3390/cryst9100544

[CR39] Sheth V, Chen X, Mettenbrink EM, Yang W, Jones MA, M’Saad O, Thomas AG et al (2023) Quantifying intracellular nanoparticle distributions with three-dimensional super-resolution microscopy. ACS Nano 17(9):8376–8392. 10.1021/acsnano.2c12808 37071747 10.1021/acsnano.2c12808 PMC10643044

[CR40] Shin HJ, Kwak M, Kwon I-H, Kim SH, Lee JY (2025) Quantification of cellular uptake of gold nanoparticles via scattering intensity changes in flow cytometry. Nanoscale Adv. 10.1039/D4NA00918E 40336583 10.1039/d4na00918e PMC12053468

[CR41] Sokolova V, Ebel JF, Kollenda S, Klein K, Kruse B, Veltkamp C, Lange CM, Westendorf AM, Epple M (2022) Uptake of functional ultrasmall gold nanoparticles in 3D gut cell models. Small. 10.1002/smll.202201167 35712760 10.1002/smll.202201167

[CR42] Srijampa S, Buddhisa S, Ngernpimai S, Sangiamdee D, Chompoosor A, Tippayawat P (2019) Effects of gold nanoparticles with different surface charges on cellular internalization and cytokine responses in monocytes. BioNanoScience 9(3):580–586. 10.1007/s12668-019-00638-8

[CR43] Sujai PT, Joseph MM, Saranya G, Nair JB, Murali VP, Maiti KK (2020) Surface charge modulates the internalization: vs. penetration of gold nanoparticles: comprehensive scrutiny on monolayer cancer cells, multicellular spheroids and solid tumors by SERS modality. Nanoscale 12(13):6971–6975. 10.1039/d0nr00809e 32202584 10.1039/d0nr00809e

[CR44] Torrano AA, Bräuchle C (2014) Precise quantification of silica and ceria nanoparticle uptake revealed by 3D fluorescence microscopy. Beilstein J Nanotechnol 5(1):1616–1624. 10.3762/bjnano.5.173 25383274 10.3762/bjnano.5.173 PMC4222454

[CR45] Torrano AA, Blechinger J, Osseforth C, Argyo C, Reller A, Bein T, Michaelis J, Bräuchle C (2013) A fast analysis method to quantify nanoparticle uptake on a single cell level. Nanomedicine 8(11):1815–1828. 10.2217/nnm.12.178 23384698 10.2217/nnm.12.178

[CR46] Van Der Zwaag D, Vanparijs N, Wijnands S, De Rycke R, De Geest BG, Albertazzi L (2016) Super resolution imaging of nanoparticles cellular uptake and trafficking. ACS Appl Mater Interfaces 8(10):6391–6399. 10.1021/acsami.6b00811 26905516 10.1021/acsami.6b00811

[CR47] Wang SH, Lee CW, Chiou A, Wei PK (2010) Size-dependent endocytosis of gold nanoparticles studied by three-dimensional mapping of plasmonic scattering images. J Nanobiotechnol. 10.1186/1477-3155-8-33 10.1186/1477-3155-8-33 PMC3236302 21167077

[CR48] Wang H, Chen B, He M, Li X, Chen P, Bin Hu (2019) Study on uptake of gold nanoparticles by single cells using droplet microfluidic chip-inductively coupled plasma mass spectrometry. Talanta 200(August):398–407. 10.1016/j.talanta.2019.03.075 31036201 10.1016/j.talanta.2019.03.075

[CR49] Xie X, Liao J, Shao X, Li Q, Lin Y (2017) The effect of shape on cellular uptake of gold nanoparticles in the forms of stars, rods, and triangles. Sci Rep. 10.1038/s41598-017-04229-z 28630477 10.1038/s41598-017-04229-z PMC5476625

[CR50] Yue J, Feliciano TJ, Li W, Lee A, Odom TW (2017) Gold nanoparticle size and shape effects on cellular uptake and intracellular distribution of SiRNA nanoconstructs. Bioconjug Chem 28(6):1791–1800. 10.1021/acs.bioconjchem.7b00252 28574255 10.1021/acs.bioconjchem.7b00252 PMC5737752

