# Supplementary material for: Uptake of gold nanoparticles in HeLa cells observed by confocal microscopy shows dependency on particle size and shape
Source: Eur Biophys J. 2025 Jun 19;55(2):229–40. doi: 10.1007/s00249-025-01769-5 (PMC13109166; doi:10.1007/s00249-025-01769-5)
Supplement: Supplementary file 1 — Supplementary file1 (DOCX 1468 KB) [file 249_2025_1769_MOESM1_ESM.docx]

**Supplementary Information**


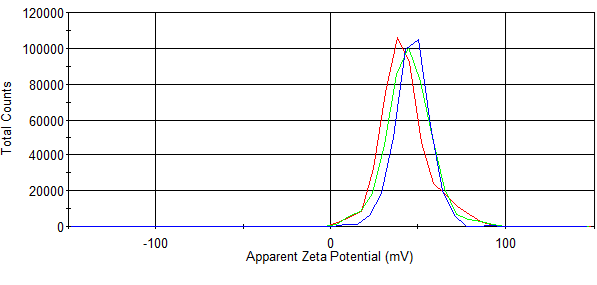

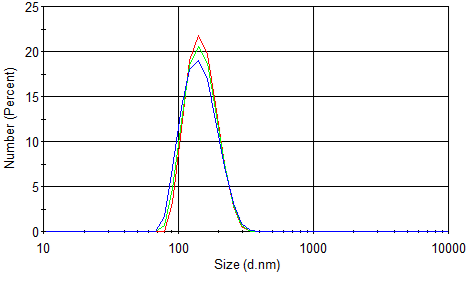

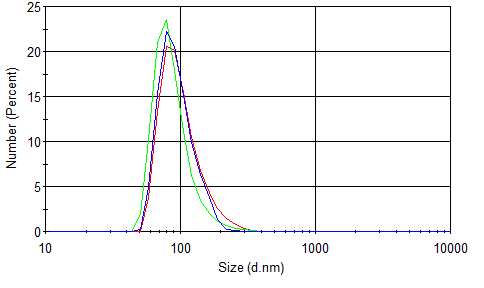


e

a

f

b

g

c


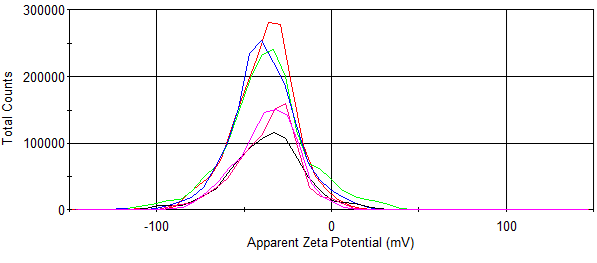


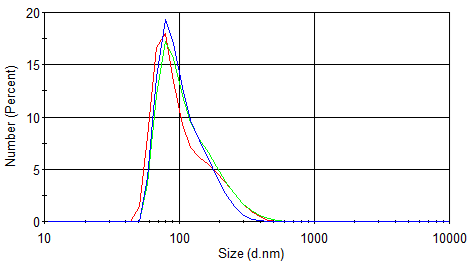


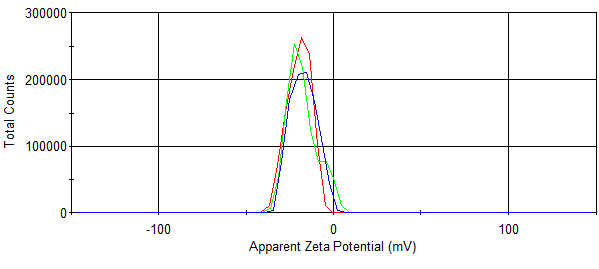


**Fig.S1:** DLS measurements on the used AuNPs: a), b), c) size and d), e), f) Zeta potential for the 150 nm spheres, 80 nm spheres and 80 nm urchins, respectively. Some deviation for the urchins in c) with respect to the nominal size of the core is observed (size peak is at 78 nm but a long tail appears at higher values, with Z-average around 100 nm). Concurrently, the Zeta potential in f) peaked around -25 mV, vs the +42 mV and the -38 mV of the nanospheres with 150 nm and 80 nm diameter, respectively.

A movie of the 3D data in Fig.1f rotating 360° in space around one axis passing through the xy plane at mid z height across the image (file MovieVertical.AVI) has been uploaded during the submission, and is available on request, which makes it easier to see which AuNPs are inside the cell.


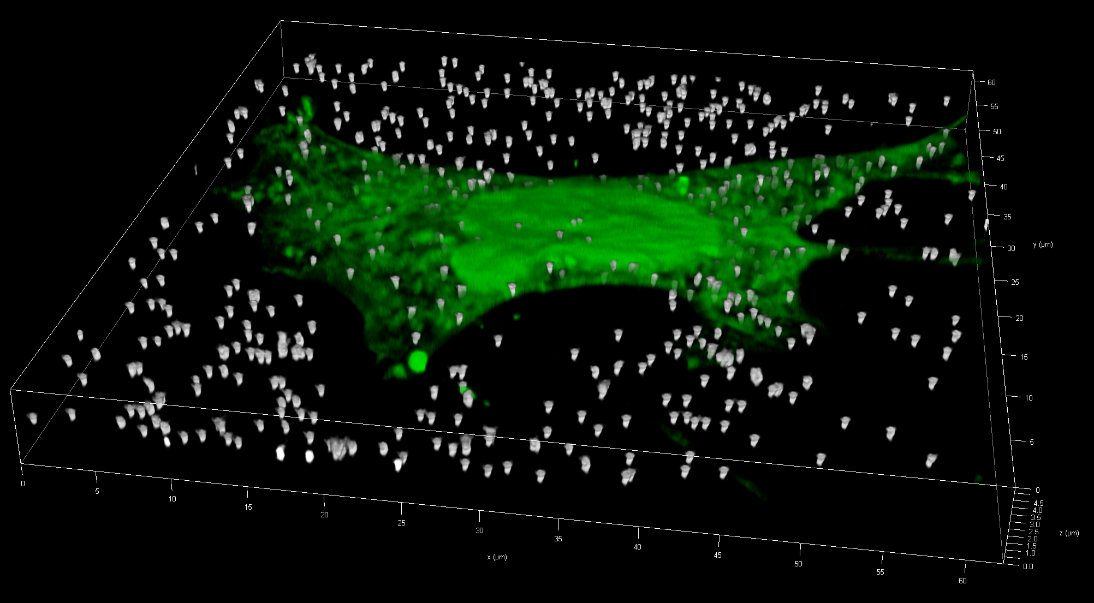


**Fig.S2:** Raw data 3D image of typical cell (green) and AuNPs (white), as obtained from the LasX acquisition software of the confocal microscope, to be compared with Fig.1f. Here, clearly the AuNPs do not occupy a single voxel, as - different from Fig.1f - the whole particle volumes are rendered and not just the position of the centroid.


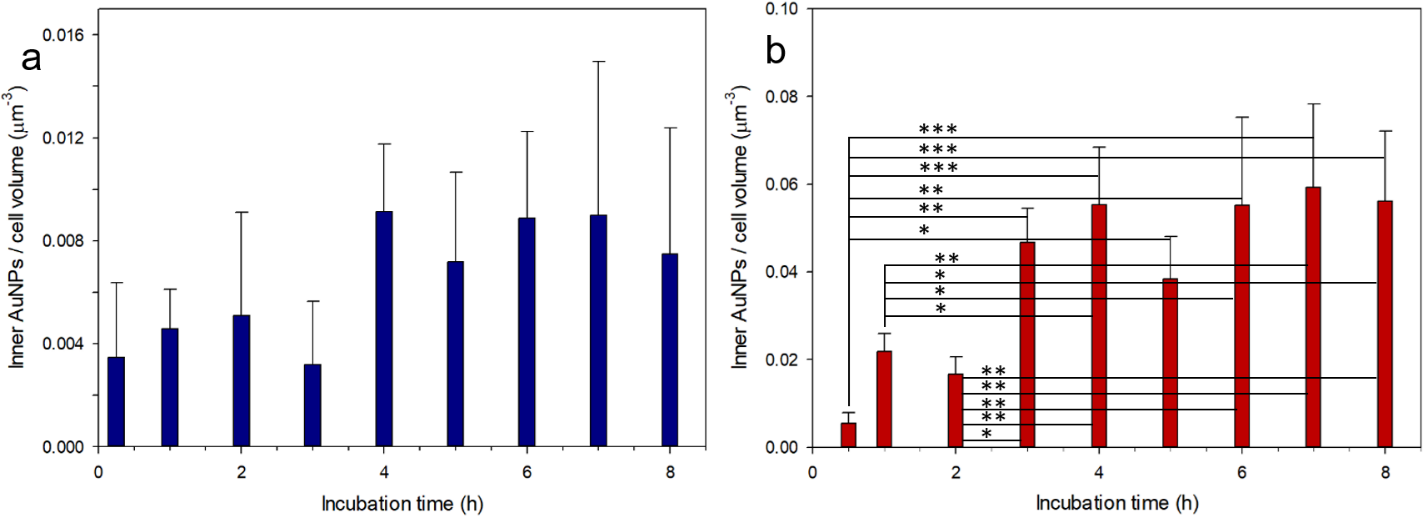


**Fig.S3:** Replicate of AuNPs internalization time profile experiment for fixed cells, with a comparison of a) 80 nm diameter urchins and b) 150 nm diameter spheres. *: statistically significance difference according to 95%, **: according to 99%, ***: according to 99.9%.


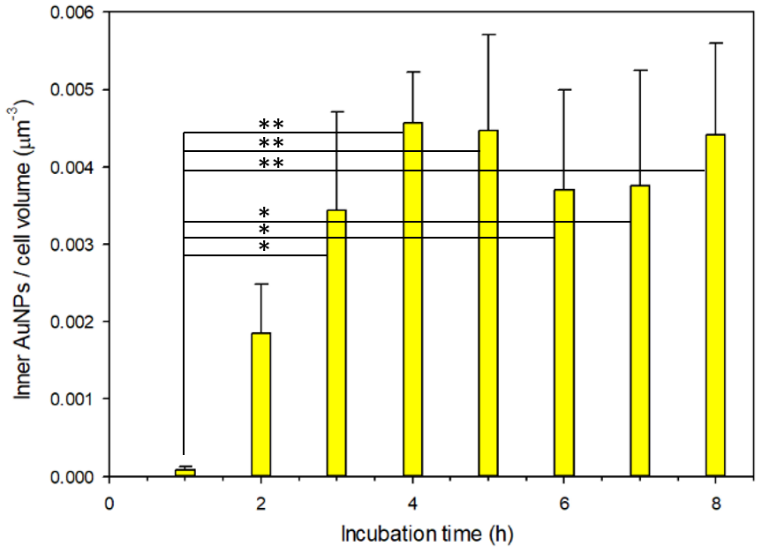


**Fig. S4:** Additional case of 100 nm spherical AuNPs, imaged in live cells (to be compared with the right column in Fig.2). Statistical significance levels: as already described in the legend to Fig.S3.

**Table S1:** MTT assay. Percentage of cell vitality following the incubation with spherical 150 nm AuNPs expressed as mean ± standard deviation.

| **Incubation time (h)  with 150 nm spherical AuNPs** | **Cell vitality  (%, mean ± std. dev.)** |
| --- | --- |
| 0 (control, without AuNPs) | 100.0 ± 6.0 |
| 1 | 105.0 ± 5.0 |
| 2 | 102.7 ± 5.7 |
| 3 | 104.3 ± 5.7 |
| 4 | 108.3 ± 5.0 |
| 5 | 108.3 ± 5.3 |
| 6 | 101.7 ± 3.7 |
| 7 | 102.0 ± 4.3 |
| 8 | 101.0 ± 6.0 |

**Table S2:** MTT assay. Percentage of cell viability after the incubation with 80 nm gold urchins expressed as mean ± standard deviation.

| **Incubation time (h)  with 80 nm gold urchins** | **Cell vitality  (%, mean ± Std. dev.)** |
| --- | --- |
| 0 (control, without AuNPs) | 100.0 ± 6.5 |
| 1 | 101.0 ± 8.5 |
| 2 | 112.0 ± 9.0 |
| 3 | 84.5 ± 7.5 |
| 4 | 107.5 ± 18.5 |
| 5 | 108.0 ± 9.5 |
| 6 | 115.5 ± 15.0 |
| 7 | 109.0 ± 11.5 |
| 8 | 106.5 ± 7.0 |

**Table S3:** Live/Dead assay. Percentage of cell viability following the incubation with spherical 150 nm AuNPs expressed as mean ± standard deviation.

| **Incubation time (h)  with 150 nm spherical AuNPs** | **Cell viability  (%, mean ± std. dev.)** | **No. of counted cells** |
| --- | --- | --- |
| 0 (control, without AuNPs) | 97.3 ± 0.7 | 7741 |
| 1 | 97.63 ± 0.88 | 8609 |
| 4 | 98.10 ± 0.22 | 11595 |
| 6 | 98.30 ± 0.72 | 13726 |
| 8 | 98.47 ± 0.25 | 14022 |
| 24 | 98.50 ± 0.39 | 16762 |

**Table S4:** Live/Dead assay. Percentage of cell viability after the incubation with 80 nm gold urchins expressed as mean ± standard deviation.

| **Incubation time (h)  with 80 nm gold urchins** | **Cell viability  (%, mean ± std. dev.)** | **No. of counted cells** |
| --- | --- | --- |
| 0 (control, without AuNPs) | 99.5 ± 0.2 | 14113 |
| 1 | 99.0 ± 0.1 | 13728 |
| 4 | 99.1 ± 0.5 | 13072 |
| 6 | 99.1 ± 0.5 | 14398 |
| 8 | 99.1 ± 0.5 | 9380 |
| 24 | 99.3 ± 0.3 | 12717 |


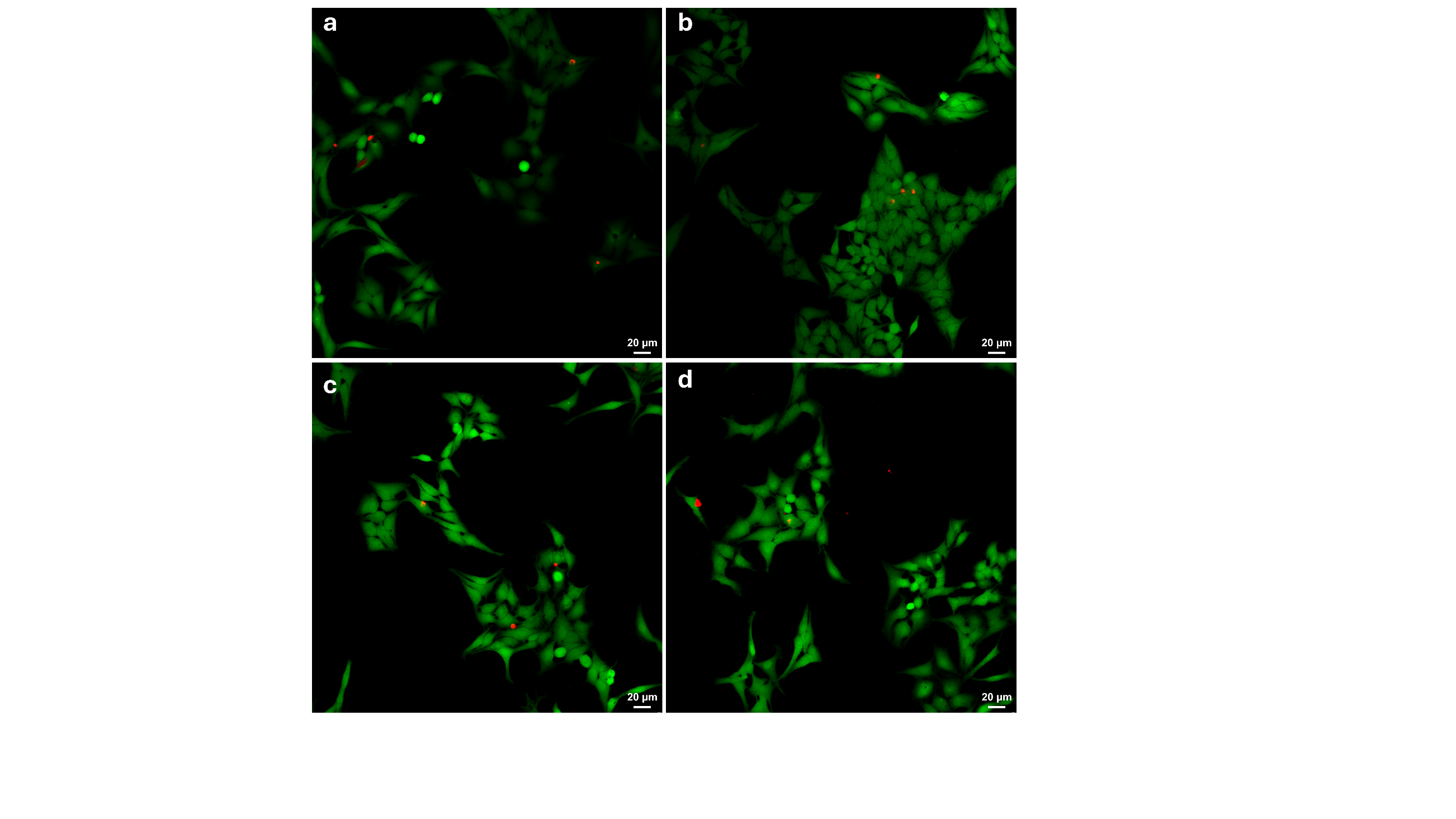


**Fig.S5:** Representative images from the Live/Dead assay showing live cells in green and dead cells in red. a) Untreated control cells and b) cells exposed to 24 h to 150 nm spherical AuNPs. c) Untreated control cells and d) cells incubated 24 h with 80 nm gold urchins.
